# Development of a Cell-penetrating Peptide that Exhibits Responsive Changes in its Secondary Structure in the Cellular Environment

**DOI:** 10.1038/srep33003

**Published:** 2016-09-09

**Authors:** Hiroko Yamashita, Takuma Kato, Makoto Oba, Takashi Misawa, Takayuki Hattori, Nobumichi Ohoka, Masakazu Tanaka, Mikihiko Naito, Masaaki Kurihara, Yosuke Demizu

**Affiliations:** 1 National Institute of Health Sciences, Tokyo 158-8501, Japan; 2 Graduate School of Biomedical Sciences, Nagasaki University, 1-14 Bunkyo-machi, Nagasaki 852-8521, Japan

## Abstract

Cell-penetrating peptides (CPP) are received a lot of attention as an intracellular delivery tool for hydrophilic molecules such as drugs, proteins, and DNAs. We designed and synthesized nona-arginine analogues **1**–**5** [FAM-β-Ala-(l-Arg-l-Arg-l-Pro)_3_-(Gly)_3_-NH_2_ (**1**), FAM-β-Ala-(l-Arg-l-Arg-l-Pro^NH2^)_3_-(Gly)_3_-NH_2_ (**2**), FAM-β-Ala-(l-Arg-l-Arg-l-Pro^Gu^)_3_-(Gly)_3_-NH_2_ (**3**), FAM-β-Ala-(l-Arg)_2_-(l-Pro^Gu^)_2_-(l-Arg)_4_-l-Pro^Gu^-(Gly)_3_-NH_2_ (**4**), and FAM-β-Ala-(l-Arg)_6_-(l-Pro^Gu^)_3_-(Gly)_3_-NH_2_ (**5**)] containing l-proline (l-Pro) or cationic proline derivatives (l-Pro^NH2^ and l-Pro^Gu^), and investigated their cell-penetrating abilities. Interestingly, only peptide **3** having the side-chain guanidinyl l-Pro^Gu^ exhibited a secondary structural change in cellular environment. Specifically, peptide **3** formed a random structure in hydrophilic conditions, whereas it formed a helical structure under amphipathic conditions. Furthermore, during cellular permeability tests, peptide **3** demonstrated greater cell-penetrating activity than other peptides and effectively transported plasmid DNA into HeLa cells. Thus, l-Pro^Gu^-containing peptide **3** may be a useful candidate as a gene delivery carrier.

l-Proline (l-Pro) is a genetically encoded amino acid. It has a variety of unique properties, and hence, has attracted great interest from chemists and biologists. From the point of view of the secondary structures of proteins, l-Pro is often found in turn structures and is considered to be a potent breaker of helical and sheet structures^1^,^2^,^3^,^4^. On the other hand, oligoprolines form a characteristic helical structure called a polyproline helix^5^,^6^, and l-Pro residues in peptide sequences sometimes induce specific secondary structural changes in an environment-dependent manner^7^,^8^. Thus, l-Pro is a useful amino acid for creating peptides with secondary structures that are able to adapt to environmental changes. To date, various conformationally restricted Pro-rich peptides have been developed as cell-penetrating peptides (CPP) and have been used as intracellular delivery tools for hydrophilic molecules^9^,^10^,^11^,^12^,^13^. Recently, we investigated how the secondary structures of CPP influence their cell-penetrating activity. Specifically, α-aminoisobutyric acid (Aib)^14^,^15^,^16^,^17^,^18^, which is a representative α,α-disubstituted amino acid (dAA) and is often used to stabilize peptide helical structures, was incorporated into an arginine (Arg)-based nonapeptide at the 3^rd^, 6^th^, and 9^th^ positions to generate the amphipathic peptide FAM-β-Ala-(l-Arg-l-Arg-Aib)_3_-NH_2_ (**A**)^19^. Conformational analyses and cell-permeability tests demonstrated that the peptide’s helical structure contributes to its ability to efficiently pass through the cell membrane. Furthermore, we recently developed a cationic dAA, Api^C2Gu^, as an Arg mimic and replaced the hydrophobic Aib residues in peptide **A** with cationic Api^C2Gu^ residues. The cationic peptide **B** also formed a stable helical structure and exhibited greater cell permeability than nona-arginine (R9)^20^. These studies suggested that the secondary structures and cell permeability of CPP are related (Fig. 1). However, previous studies examining the development of CPP have focused on stabilizing the peptides’ secondary structures in all environments^9^,^10^,^11^,^12^,^13^,^20^,^21^,^22^,^23^,^24^,^25^,^26^,^27^. On the other hand, proteins change their secondary structures in response to subtle environmental changes in the living body. These dynamic conformational changes contribute to the versatile functional expression of proteins, and therefore, conformational flexibility is a key factor in functional diversification, effective functional expression, and functional improvement. So, it is considered that a new peptide with the ability to change its secondary structure depending on its environment would exhibit a greater ability to penetrate the cell-membrane. Herein, we speculated that the insertion of an l-Pro analogue with a guanidinylated side chain into Arg-based peptides could result in a peptide with a secondary structure that was able to adapt to the surrounding environment.

In this study, we designed five types of cationic peptide (**1**–**5**): FAM-β-Ala-(l-Arg-l-Arg-l-Pro)_3_-(Gly)_3_-NH_2_ (**1**), which contained an l-Pro residue; FAM-β-Ala-(l-Arg-l-Arg-l-Pro^NH2^)_3_-(Gly)_3_-NH_2_ (**2**), which contained an l-Pro^NH2^ residue bearing a side-chain amino group; and FAM-β-Ala-(l-Arg-l-Arg-l-Pro^Gu^)_3_-(Gly)_3_-NH_2_ (**3**), which had an l-Pro^Gu^ residue bearing a side-chain guanidino group. Furthermore, FAM-β-Ala-(l-Arg)_2_-(l-Pro^Gu^)_2_-(l-Arg)_4_-l-Pro^Gu^-(Gly)_3_-NH_2_ (**4**) and FAM-β-Ala-(l-Arg)_6_-(l-Pro^Gu^)_3_-(Gly)_3_-NH_2_ (**5**), which are isomeric sequences of peptide **3**, were designed (Fig. 2). As mentioned above, the incorporation of l-Pro residues has various effects on the secondary structures of peptides so it was considered that the synthesized peptides might display interesting environment-dependent structural changes. We analyzed the peptides’ preferred secondary structures under hydrophilic and amphipathic conditions based on their CD spectra and assessed their intracellular permeability using adhesive and non-adhesive cells. We also conducted an experiment in which the transportation of pDNA into HeLa cells was examined in the presence of the peptides.

## Results and Discussion

Two types of Fmoc-protected Pro derivatives, Fmoc-l-Pro^NH2^-OH and Fmoc-l-Pro^Gu^-OH were synthesized prior to the solid-phase synthesis of the peptides according to the previously reported synthetic routes^28^,^29^. Then, Fmoc-l-Pro-OH, Fmoc-l-Pro^NH2^-OH, and Fmoc-l-Pro^Gu^-OH were incorporated into R9 analogues to afford the desired peptides (**1**–**5**), all of which contained N-terminal fluorescein (FAM) labels and C-terminal Gly_3_ linkers (Gly linkers were inserted for efficient Pro^Gu^-containing peptide synthesis). These peptides were synthesized by Fmoc-based solid-phase methods and purified by reversed-phase high performance liquid chromatography.

**Figure 3:**
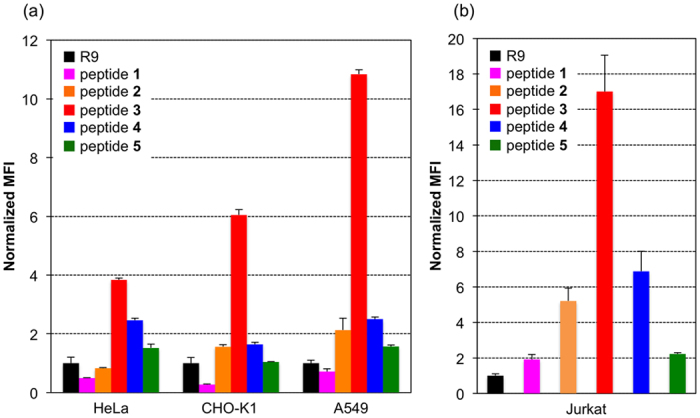
The intracellular uptake of peptides **1**–**5** and R9 by (**a**) adhesive cells (HeLa, CHO-K1, A549) and (**b**) non-adhesive Jurkat cells. Mean fluorescence intensity of the cells normalized to R9. The cells were incubated with 1 μM peptides for 2 hr and their intracellular fluorescence was measured by flow cytometry. Values are the means ± standard deviation of three independent cultures.

First, we examined the ability of peptides **1**–**5** and R9 to cross the cell membrane using adhesive HeLa, CHO-K1, and A549 cells, and non-adhesive Jurkat cells, by flow cytometry. The cells were treated with 1 μM of the relevant peptide and incubated for 2 h at 37 °C. Then, the mean fluorescence intensity of the cells was measured. Figure 3 shows the intracellular uptake efficiency of peptides **1**–**5** and R9. In a comparison of the cell-penetrating activities of peptides **1**–**5**, all of which had l-Pro-containing skeletons, peptide **3**, which possessed a guanidinylated side chain, exhibited extremely high activity in both the adhesive and non-adhesive cells. These results matched those of our recent report, which found that cationic functional groups, especially side-chain guanidino groups, enhance cellular internalization^20^. However, the cell-penetrating activity of peptide **3** was 2.5 to 17 times higher than that of R9, which contains the same number of guanidino groups in its sequence. In addition, this peptide transferred into the cell with higher efficiency in comparison to peptides **4** and **5** containing the same number of l-Arg and l-Pro^Gu^ residues. Therefore, besides the effects of its side-chain guanidine group, other factors might increase the cell-penetrating ability of peptide **3**. Furthermore, in our recent study, no significant differences were observed between the cell-penetrating activities of an amino-type helical peptide composed of three amino and six guanidino groups and a guanidine-type helical peptide containing nine guanidino groups^20^. Thus, we analyzed the preferred secondary structures of the synthesized peptides by measuring their CD spectra in various conditions to investigate the differences in their cellular permeability from the point of view of their secondary structures.

**Figure 4:**
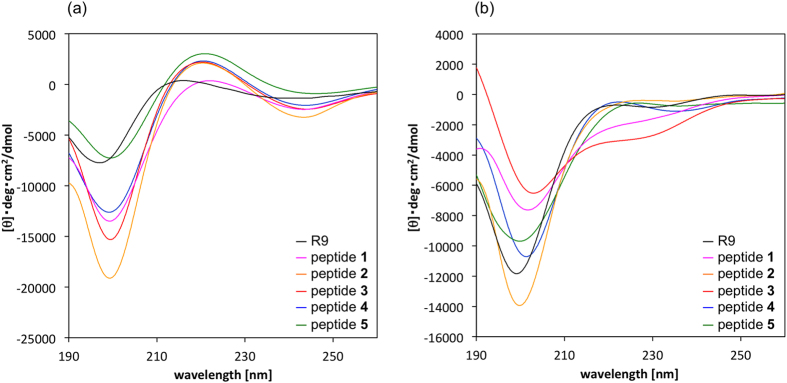
CD spectra of peptides **1**–**5** and R9 in (**a**) 20 mM phosphate buffer (pH = 7.4), and (**b**) 1.0 w/v% SDS in phosphate buffer. Peptide concentration: 100 μM.

Figure 4 shows the CD spectra of peptides **1**–**5** and R9 in 20 mM phosphate buffer solution and in 1.0 w/v% sodium dodecyl sulphate (SDS) solution. The spectra of all of the peptides showed negative maxima at around 200 and 245 (weak) nm and weak positive maxima at around 220 nm, indicating that those peptides formed random structures in phosphate buffer (under physiological conditions) (Fig. 4a)^8^. Whereas, marked conformational changes were observed in peptide **3** in SDS solution, which simulates the environment found near the cell membrane (Fig. 4b)^20^,^30^,^31^,^32^. Specifically, under amphipathic conditions the spectrum of peptide **3** exhibited negative maxima at around 205 and 225 nm, indicating that the preferred secondary structure of **3** changed from a random structure to a helical structure^33^,^34^,^35^,^36^. The ideal α-helical peptide such as peptide **B** shows negative maxima at around 208 and 222 nm in the spectrum, and therefore, peptide **3** might not form an α-helical but a helix-like structure (Fig. S7). Accordingly, peptide **3** might be able to form a helical structure and become more compact in the cell membrane environment, and the greater cell-penetrating ability of peptide **3** might arise from these specific conformational changes. On the other hand, peptides **4** and **5**, which had as many Arg and Pro^Gu^ as peptide **3** but these amino acids were not arranged regularly, didn’t show the spectra specific to helical structure. These results indicated that regular array of amino acids induces organized secondary structures. l-Pro residues in peptide sequences sometimes induce specific secondary structural changes from random to helical structures in an environment-dependent manner^8^. In the reference, in fact, the helical propensity of Pro was found to be greatly enhanced in the membrane-mimetic environment (SDS in buffer), analyzing by their CD spectra. Thus, the peptide **3** might also form a random structure in hydrophilic conditions (in PBS), and change its secondary structure to a helical structure under amphipathic conditions (1% SDS in PBS). Whereas, the preferred conformations of peptides **4** and **5**, which are isomeric sequences of peptide **3**, were almost no changes in hydrophilic/amphipathic conditions. Furthermore, compared to the cell-penetrating activities of these three isomeric peptides **3**–**5**, the activity of peptide **3** was superior to those of peptides **4** and **5**. Considering the relationship of the conformations and activities of **3**–**5**, the high cell-penetrating activity of peptide **3** is possible to result from the secondary structure in a certain environment.

**Figure 5:**
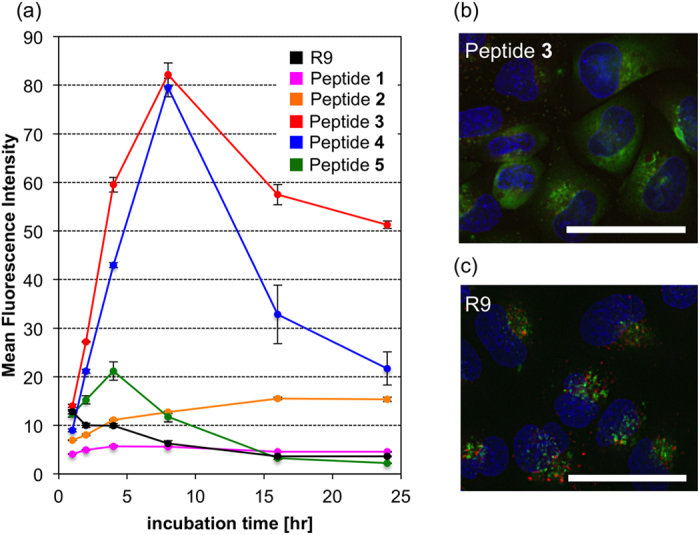
(**a**) Cellular uptake of the peptides **1**–**5** and R9 after 1–24 hr (peptide concentration: 1 μM). Values are the means ± standard deviation of three independent cultures. (**b,c**) Peptide **3** and R9 were colocalized with lysosome marker. HeLa cells were treated with 10 μM peptides at 37 °C for 2 hr. The acidic late endosomes/lysosomes were stained with LysoTracker Red (red), and the nuclei were stained with Hoechst 33342 (blue). The scale bars represent 50 μm.

Since peptide **3** exhibited a superior cell-penetrating ability, which led us to focus on its utility as an intracellular delivery tool, we evaluated the stability and cytotoxicity of the peptides. Figure 5a shows the uptake of peptides **1**–**5** and R9 by HeLa cells during their incubation at 37 °C for 1–24 hr. The cellular uptake of R9 gradually decreased after 1-hr incubation, indicating that R9 is unstable in culture medium containing fetal bovine serum (FBS). Whereas, the cellular uptake of l-Pro-containing **1** and l-Pro^NH2^-containing **2** did not change much over 1-24-hr incubation, but exhibited lower fluorescence intensity than l-Pro^Gu^-containing peptides **3** and **4** at 1-24-hr points. In contrast, the uptake of **3** and **4** gradually increased from 1 to 8-hr incubation, suggesting that these peptides were more stable than R9 in the cellular environment. However, the peptide **5**, containing the same number of l-Pro^Gu^ residues as peptide **3**, didn’t show durable permeability. These results indicated that an insertion point of non-proteinogenic amino acid has an effect on the peptides’ stability in the medium. The peptides’ stability was also analyzed by the following two methods: Figure S8a shows the uptake of R9 and peptides **1**–**5** by HeLa cells after the peptides had been exposed to the medium containing 10% FBS for 0–24 h at 37 °C, and were then incubated with the cells for 2 hr at 37 °C. Figure S8b shows the peptides’ stability (1–24 hr) in culture medium containing 10% FBS using LC-MS analysis. Considering the pre-incubation experiment and LC-MS analysis, the cell-penetrating abilities and stabilities of R9 and L-Pro-containing **1** sharply fell as the pre-incubation time increased, compared with those of l-Pro^NH2^-containing **2** and l-Pro^Gu^-containing **3**. These results also indicated that peptides **2** and **3** are more chemically stable than R9 and peptide **1**. The results of the cytotoxicity analysis, in which HeLa cells were treated with peptides **1**–**5** for 24 hr at concentrations of 1, 4, and 8 μM, are shown in Fig. S9. None of the peptides exhibited significant cytotoxicity under these experimental conditions, indicating the low cellular toxicity of each peptide and the possibility of these peptides as carrier peptides for hydrophilic molecules. Next, we investigated the intracellular uptake pathways of R9 and peptide **3**. In the presence of various endocytosis inhibitors (amiloride, a macropinocytosis inhibitor^37^; nystatin, a caveolae-mediated endocytosis inhibitor^38^; and sucrose, a clathrin-mediated endocytosis inhibitor^39^), the migration levels of R9 and **3** into the HeLa cells were compared (Fig. S10). The uptake of R9 was decreased by treatment with amiloride or sucrose, indicating that R9 passes through the cell membrane via macropinocytosis and clathrin-mediated endocytosis. On the other hand, the uptake of **3** was slightly inhibited by treatment with amiloride (approximately 20% inhibition), indicating that macropinocytosis is one of the uptake pathways of **3** and the other pathways may be also present. Then, we investigated the intracellular localization and dynamic behavior of R9 and **3** using fluorescence microscopy. The cells’ late endosomes/lysosomes were stained with LysoTracker Red (red), and their nuclei were stained with Hoechst 33342 (blue). The results are shown in Fig. 5b,c. Surprisingly, the escape of peptide **3** from endosomes was observed after 30-min incubation (Fig. S11). After 2 hr, some cells did not display any endosome-like small green spots (Fig. 5b). On the other hand, small green spots were observed in the cells and co-localized with the late endosomes/lysosomes incubated with R9 or peptide **2** even after 2-hr incubation (Figs 5c and S12). These results indicate that peptide **3** is more capable of escaping from endosomes than R9 and peptide **2**, and exists in cytosol. As a general approach, peptides that can change their helical structures in accordance with pH fluctuations are often used to promote escape from endosomes^40^,^41^,^42^,^43^. Therefore, it is assumed that the specific conformational changes exhibited by peptide **3** (from a random structure to a helical structure) contribute to its effective escape from endosomes. We considered that peptide **3** might also directly penetrate the cell membrane more efficiently than R9 (R9 passes through the cell membrane via not only endocytosis but also non-endocytosis pathway, that is, direct permeation to the cell membrane)^44^. Therefore, we investigated the intracellular uptake of peptide **3** at low temperature using flow cytometry and fluorescence microscopy. At low temperature, energy-dependent pathways such as endocytosis are inhibited so it is possible to assess direct peptide penetration via energy-independent pathways. From fluorescence microscopy images obtained under low-temperature conditions, it was determined that peptide **3** was able to pass through the cell membrane directly via a pathway other than endocytosis (Fig. S13a,b). Interestingly, unlike R9 (Fig. S13c), peptide **3** localized in the cytosol and specific organs in the nucleus. Moreover, the direct penetration of **3** was also confirmed using liposomes (Fig. S13d). Peptides that can directly penetrate through the cell membrane can be used for efficient intracellular delivery as they are not affected by problems associated with the need to escape from endosomes.

**Figure 6:**
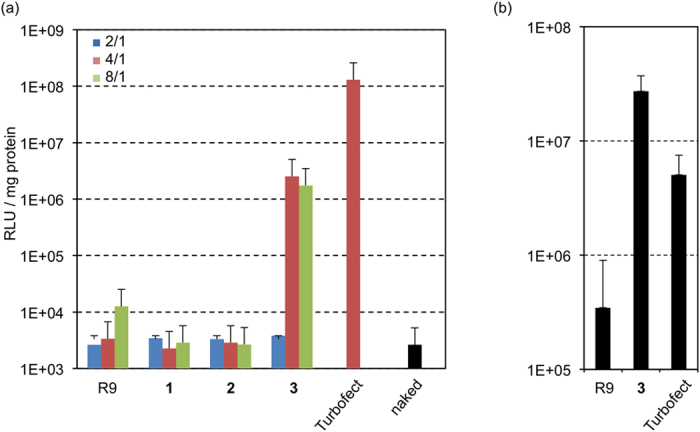
Transfection efficiency of **1**–**3** and R9/pDNA complexes at (**a**) 24-hr and (**b**) 48-hr post-incubation in HeLa cells. Data are shown as the mean ± standard deviation values of three independent cultures.

Finally, we conducted pDNA intracellular transport experiments using peptides **1**–**3**, R9, and HeLa cells. Peptide/pDNA complexes were prepared at charge ratios of 2/1, 4/1, and 8/1 because the residual molar ratios of the amino and/or guanidino groups in the peptides have to correspond to the number of phosphate groups in the pDNA. The transfection efficiency of peptides **1**–**3** and R9 was assessed using a luciferase-based assay. Figure 6 shows the transfection efficiencies of these peptides. pDNA transfection efficiency of the synthesized peptides were lower than that of commercially available transfection reagent TurboFect at 24-hr post-incubation, and peptide **3** transported the pDNA into both types of cells more efficiently than other peptides (Fig. 6a). However, peptide **3**/pDNA (8/1) complex reached the higher transfection efficiency at 48-hr post-incubation than TurboFect/pDNA (4/1) complex did (Fig. 6b), indicating that peptide **3** was resistant to enzymatic degradation by proteases in cells, and therefore, appeared to have prolonged transfection abilities due to the protection of encapsulated pDNA in complexes for a longer time (Detailed physicochemical properties and transfection mechanism of peptide/pDNA complexes were most recently reported by our group)^45^.

In summary, we designed and synthesized five types of cationic peptide; i.e., FAM-β-Ala-(l-Arg-l-Arg-l-Pro)_3_-(Gly)_3_-NH_2_ (**1**), FAM-β-Ala-(l-Arg-l-Arg-l-Pro^NH2^)_3_-(Gly)_3_-NH_2_ (**2**), and FAM-β-Ala-(l-Arg-l-Arg-l-Pro^Gu^)_3_-(Gly)_3_-NH_2_ (**3**), FAM-β-Ala-(l-Arg)_2_-(l-Pro^Gu^)_2_-(l-Arg)_4_-l-Pro^Gu^-(Gly)_3_-NH_2_ (**4**), and FAM-β-Ala-(l-Arg)_6_-(l-Pro^Gu^)_3_-(Gly)_3_-NH_2_ (**5**). Permeability tests of peptides **1**–**5** and R9 showed that peptide **3**, in which guanidinylated proline residues were arranged in regular positions (3^rd^, 6^th^, and 9^th^), had a much greater cell-penetrating ability. The cell-penetrating activity of **3** was greater than that of R9 and peptides **4**–**5**, suggesting that this superior activity results from the specific secondary structure of **3**. Therefore, we analyzed the preferred secondary structures of peptides **1**–**5** and R9 by measuring their CD spectra. As a result, it was revealed that **3** formed a helical structure as its preferred conformation in amphipathic conditions, whereas it formed a random structure in phosphate buffer solution. We considered that these specific conformational changes contribute to the greater cell-penetrating activity of **3**. Moreover, in order to evaluate the utility of the peptides as intracellular delivery tools, we examined the stability, cytotoxicity, and endosomal escape functions of peptides **1**–**5** and R9. Accordingly, it was confirmed that l-Pro^Gu^-containing peptide **3** remained stable in the medium containing FBS, and none of the peptides exhibited cytotoxicity. Moreover, **3** seems to escape from the endosomes more efficiently than R9 and **2**. So, finally we conducted a plasmid DNA transportation experiment using peptides **1**–**3** and R9 to evaluate the transfection efficiencies of these peptides. As had been expected, **3** transported pDNA into HeLa cells more efficiently than R9, suggesting that **3** would be useful as a carrier peptide for transporting hydrophilic molecules.

## Methods

### Synthesis and characterization of N-terminal-protected amino acids and peptides

Fmoc-l-Pro^NH2^-OH and Fmoc-l-Pro^Gu^-OH were synthesized prior to the solid-phase synthesis of the peptides according to the previously reported synthetic routes^28^,^29^. Peptides were synthesized on a solid support using Fmoc solid-phase methods with standard commercially available Rink amide resin and Fmoc-amino acids. Detailed experimental procedures, HPLC charts, and mass spectrometric data of each peptide were shown in Supplementary Data.

### CD spectrometry

CD spectra were recorded with a *Jasco J-720W* spectropolarimeter using a 1.0 mm path length cell. The data are expressed in terms of [θ]; i.e., total molar ellipticity (deg cm^2^ dmol^−1^). 20 mM phosphate buffer (pH = 7.4) and 1% SDS in 20 mM phosphate buffer (pH = 7.4) were used as solvents. Peptide concentration; 100 μM.

### Cellular uptake of peptidesh

HeLa, A549, Jurkat and CHO-K1 cells were seeded in 6-well dishes at a density of 4.0 × 10^6^ cells/well and cultured in DMEM (HeLa, A549), RPMI-1640 (Jurkat) and Ham’s F-12 (CHO-K1) for 24 hr, respectively. The cells were treated with each peptide (peptide concentration; 1 μM) and incubated for each time (1, 2, 4, 8, 16 and 24 hr). Then, the cells were washed three times with phosphate buffer (PBS) supplemented with heparin (20 units/mL) and detached by treatment of trypsin-EDTA. The collected cells were pelleted by centrifugation at 3000 rpm for 5 min and the supernatant was removed. The cells were washed twice with PBS buffer. Then, the collected cells were suspended in 500 μL of PBS buffer and mean fluorescence intensity in cells was measured by flow cytometer. The results are presented as the mean and standard deviation obtained from 3 samples.

### Cytotoxicity of peptides

HeLa cells were seeded onto 96-well culture plate (2500 cells/well) and incubated for 24 hr in DMEM containing 10% FBS. The medium was replaced and peptide solution in fresh DMEM was added at each concentration (1, 4, 8 μM). After 24 h, cell viability was evaluated using cell counting kit-8 (DOJINDO) following to the manufacture’s protocol. The results are presented as the mean and standard error values obtained from 4 samples. Statistical differences were analyzed by Student’s t-test.

### Inhibition of endocytosis

The cells were seeded onto 6-well culture plates (400,000 cells/well) and incubated overnight in 2 mL of DMEM containing 10% FBS. After the medium had been replaced with fresh medium containing 10% FBS in the absence or presence of amiloride (25 μM), sucrose (0.4 M), or nystatin (25 μg/mL), the cells were pre-incubated at 37 °C for 30 min. Peptide solution was applied to each well at a concentration of 1 μM. After the cells had been incubated for 1 hr, the medium was removed, and the cells werde washed 3 times with PBS supplemented with heparin (20 units/mL) and detached by treatment of trypsin-EDTA. Then, fluorescence intensity in the cells was measured as above. The results are presented as the mean and standard error values obtained from 3 samples. Statistical differences were analyzed by Student’s t-test.

### Fluorescence microscope

HeLa cells were seeded onto glass bottom dish (Greiner Bio-one, Tokyo, Japan) (10,000 cells/well) and incubated overnight in 2 mL of DMEM containing 10% FBS. The medium was then replaced with fresh medium containing 10% FBS, and peptide solution was applied to well at a concentration of 10 μM. After the cells had been incubated for 15 min-2 hr or 30 min at 4 °C, the medium was removed, and the cells were washed 3 times with ice-cold PBS supplemented with heparin (20 units/mL). The intracellular distribution of the complexes was observed by MFM after staining late endosomes/lysosomes with LysoTracker Red and nuclei with Hoechst 33342. The MFM observations were performed using a BZ-9000 (Keyence, Osaka, Japan) equipped with a 40X objective lens.

### Confocal laser microscope of liposomes

Egg-yolk phosphatidylcholine, egg-yolk phosphatidylglycerol, and egg-yolk phosphatidylethanolamine were dissolved in chloroform (molar rate 2/2/1, total 1 μmol) and the resulting solution was evaporated to a small volume under a stream of N_2_ to form thin film. Then, the film was dried under reduced pressure for over 6 hr. After the drying, buffer **A** (10 mM Tris-HCl, 50 mM NaCl, and 10 mM sucrose, pH = 7.4) was added to the film slowly and hydrated for 2 days at rt. The resulting solution was collected and centrifuged at 10,000 rpm for 30 min at 10 °C to give the giant unilamellar vesicles (GUV). The 2 μM peptide solution (100 μL) in buffer **B** (10 mM Tris-HCl, 50 mM NaCl, and 10 mM glucose, pH = 7.4) was added to 100 μL GUV solution in buffer **A** and observed using confocal laser microscope.

### Intracellular delivery of plasmid DNA

HeLa cells were separately seeded onto 24-well culture plates (10,000 cells/well) and incubated overnight in 400 μL of DMEM containing 10% FBS. The medium was exchanged, and the peptide/pDNA complex solutions (33.3 μg pDNA/mL) prepared at various charge ratio (2/1, 4/1, 8/1), TurboFect (commercially available transfection reagent)/pDNA (at various charge ratio 8/1), and naked pDNA were applied to each well. The amount of pDNA was adjusted to 1 μg per well. After 24-hr incubation, the medium was replaced with 400 μL of fresh medium, followed by incubation. Luciferase gene expression was then evaluated based on photoluminescence intensity using the Luciferase assay kit and a Luminometer (Gene Light GL-210A, Microtec. Co., Ltd., Chiba, Japan). The amount of protein in each well was concomitantly determined using a Micro BCA protein assay kit. The results are presented as the mean and standard deviation obtained from 4 samples.

## Additional Information

**How to cite this article**: Yamashita, H. *et al*. Development of a Cell-penetrating Peptide that Exhibits Responsive Changes in its Secondary Structure in the Cellular Environment. *Sci. Rep.* **6**, 33003; doi: 10.1038/srep33003 (2016).

## Supplementary Material

Supplementary Information

## Figures and Tables

**Figure 1:**
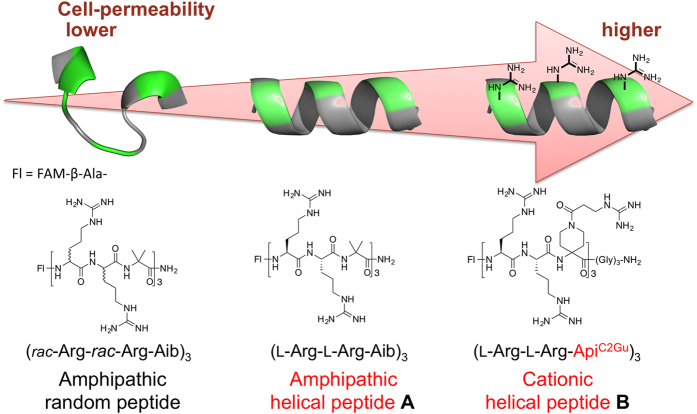
Relationship between the secondary structures of Arg/dAA-based CPP, and their cell-penetrating abilities.

**Figure 2:**
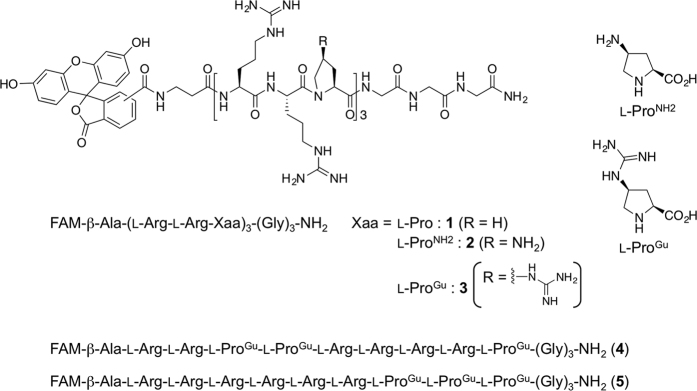
Chemical structures and sequences of peptides 1–5.
