# Supplementary material for: Development of a Cell-penetrating Peptide that Exhibits Responsive Changes in its Secondary Structure in the Cellular Environment
Source: Sci Rep. 2016 Sep 9;6:33003. doi: 10.1038/srep33003 (PMC5016780; doi:10.1038/srep33003)
Supplement: Supplementary Information [file srep33003-s1.pdf]

Development of a Cell-penetrating Peptide that Exhibits Responsive Changes in its Secondary Structure in the Cellular Environment

Hiroko Yamashita,[1] Takuma Kato,[2] Makoto Oba,[2] Takashi Misawa,[1] Takayuki Hattori,[1]

Nobumichi Ohoka,[1] Masakazu Tanaka,[2] Mikihiko Naito,[1]

Masaaki Kurihara,[1]* and Yosuke Demizu[1]*

[1]National Institute of Health Sciences, Tokyo 158-8501, Japan

[2]Graduate School of Biomedical Sciences, Nagasaki University, 1-14 Bunkyo-machi, Nagasaki 852-8521, Japan

*Corresponding author

E-mail: demizu@nihs.go.jp; masaaki@nihs.go.jp

Table of contents

General	S3
Synthesis and purification of peptide **1—5** and R9	S4
Characterization of peptides (Figures S1-S6)	S5
CD spectra of peptides (Figure S7)	S9
Pre-incubation and LC-MS analysis (Figure S8)	S10
Cytotoxicity of R9 and peptides (Figure S9)	S10
Effect of endocytosis inhibitors (Figure S10)	S11
Fluorescence microscopy images (Figures S11 and S12)	S11
Direct cell permeability of peptides (Figure S13)	S13

General. All coupling reagents were obtained from *Watanabe Chemical Industries, LTD*. and were used as supplied without further purification. Fmoc-protected amino acids were obtained from *Tokyo Chemical Industry Co., LTD*. and *Watanabe Chemical Industries, LTD.* Analytical thin-layer chromatography was performed on *Merck* Silica Gel F_{254}. High-resolution mass spectra were recorded with *SHIMAZU* LCMS-IT-TOF spectrometer. The purified peptides were characterized using 4800 Plus MALDI TOF/TOF™ Analyzer (Applied Biosystems/MDS SCIEX), and liquid chromatography–mass spectrometry-ion trap-time-of-flight (LCMS-IT-TOF) spectroscopy (Shimadzu). Fluorescence intensity was recorded with FACSCalibur (Becton Dickinson Co., Ltd.).

Synthesis and purification of peptide 1—5 and R9. The proline derivatives Fmoc-Pro^{NH2}-OH and Fmoc-Pro^{Gu}-OH were synthesized following the reported synthetic methods. The peptides were synthesized using solid-phase methods on NovaPEG Rink amide resin following the standard Fmoc chemistry. The following describes a representative coupling and deprotection cycle at a 25 μmol scale. First, 65 mg NovaPEG Rink amide resin (loading: 0.5 mmol/g) was soaked for 1 hr in CH_2Cl_2. After the resin had been washed with *N,N*-dimethylformamide (DMF), Fmoc-amino acid (4 Eq), 1-[bis(dimethylamino)methylene]-1H-1,2,3-triazolo[4,5-b]pyridinium 3-oxide hexafluorophosphate (HATU) (4 Eq) and 1-hydroxy-7-azabenzotriazole (HOAt) (4 Eq) dissolved in 1 mL *N*-methyl-2-pyrrolidone (NMP) were added to the resin. Then, *N*,*N*-diisopropylethylamine (8 Eq) was added for the coupling reaction

and the resulting mixture was shook for 1 hr at room temperature. Fmoc-deprotection was carried out by 2 mL of 20% piperidine in NMP (2 mL) for 15 min at room temperature. After the peptide elongation, the resin was suspended in cleavage cocktail [1.9 mL trifluoroacetic acid (TFA), 50 μL water, 50 μL triisopropylsilane; final concentration: 95% TFA, 2.5% water, 2.5% triisopropylsilane] for 3 h at room temperature. The TFA solution was evaporated to a small volume under a stream of N_2 and dripped into cold ether to precipitate the peptides. The dried crude peptides were dissolved in 1.3 mL of 50% acetonitrile in water and then purified by reversed-phase HPLC using a Discovery® BIO Wide Pore C18 column (25 cm x 21.2 mm). After being purified, the peptide solutions were lyophilized. Peptide purity was assessed using analytical HPLC and a Discovery® BIO Wide Pore C18 column (25 cm x 4.6 mm; solvent A: 0.1% TFA/water, solvent B: 0.1% TFA/MeCN, flow rate: 1.0 mL•mL^{-1}, gradient: 0-100% gradient of solvent B over 30 min). The peptides were characterized by matrix-assisted laser desorption/ionization mass spectrometry and LC-MS.

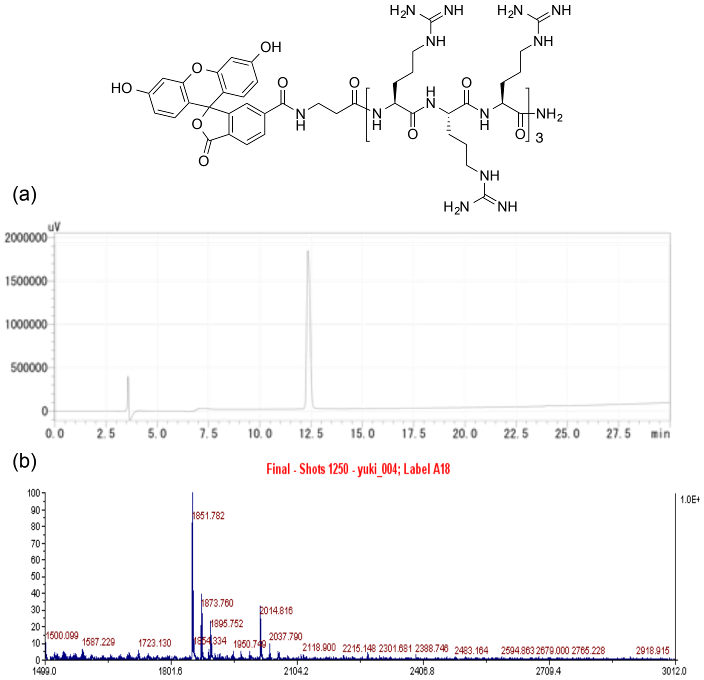

Figure S1. Purification of R9. (a) RP-HPLC profile. (b) MALDI-MS.

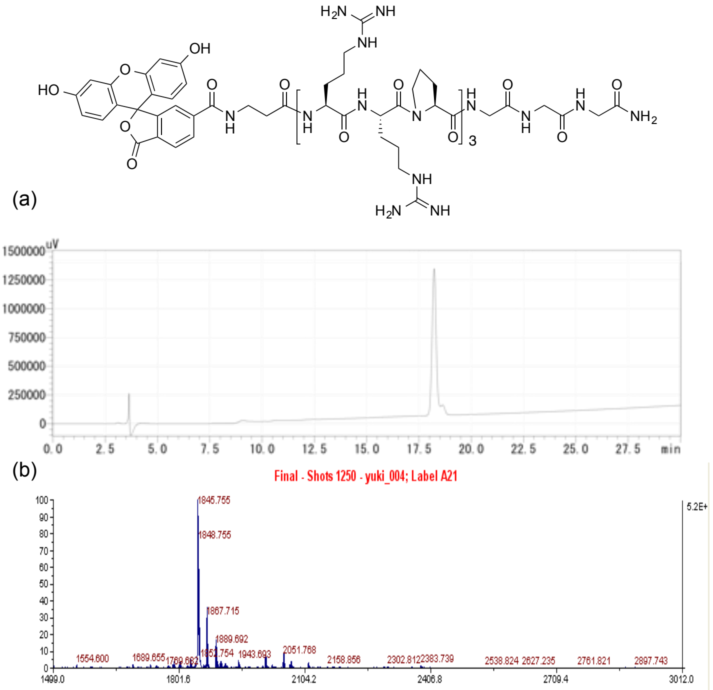

Figure S2. Purification of peptide **1**. (a) RP-HPLC profile. (b) MALDI-MS.

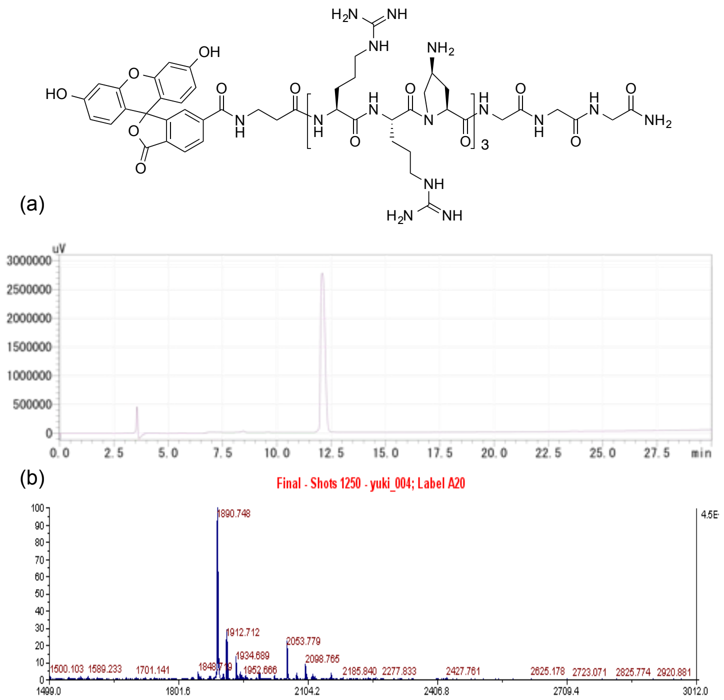

Figure S3. Purification of peptide **2**. (a) RP-HPLC profile. (b) MALDI-MS.

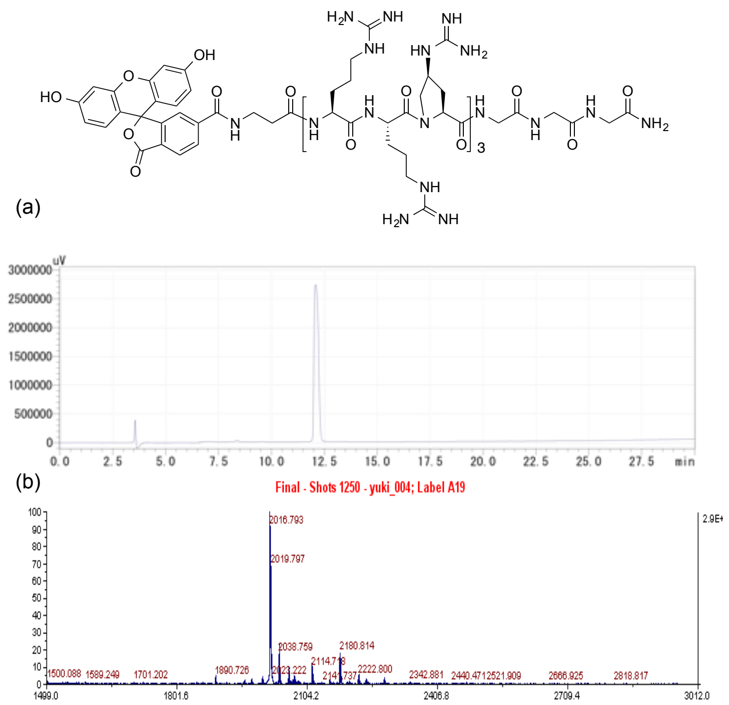

Figure S4. Purification of peptide **3**. (a) RP-HPLC profile. (b) MALDI-MS.

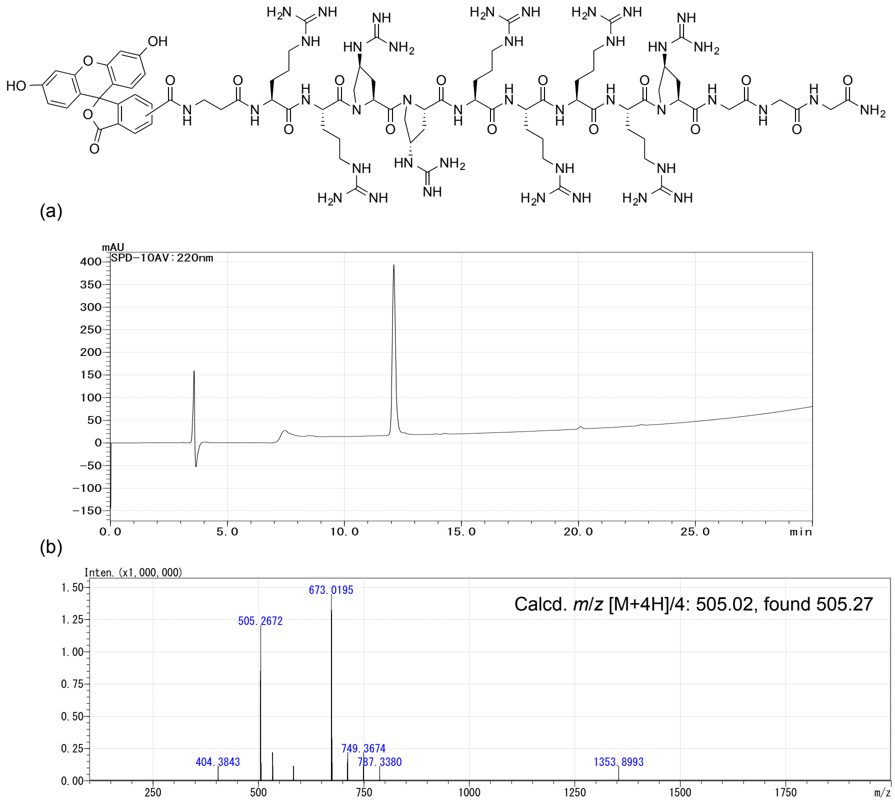

Figure S5. Purification of peptide **4**. (a) RP-HPLC profile. (b) LC-MS.

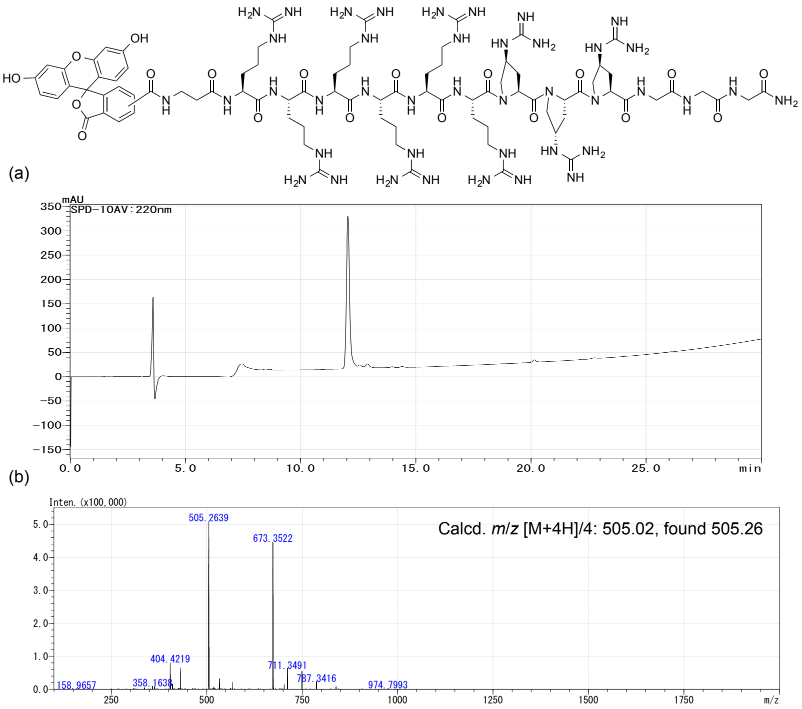

Figure S6. Purification of peptide **5**. (a) RP-HPLC profile. (b) LC-MS.

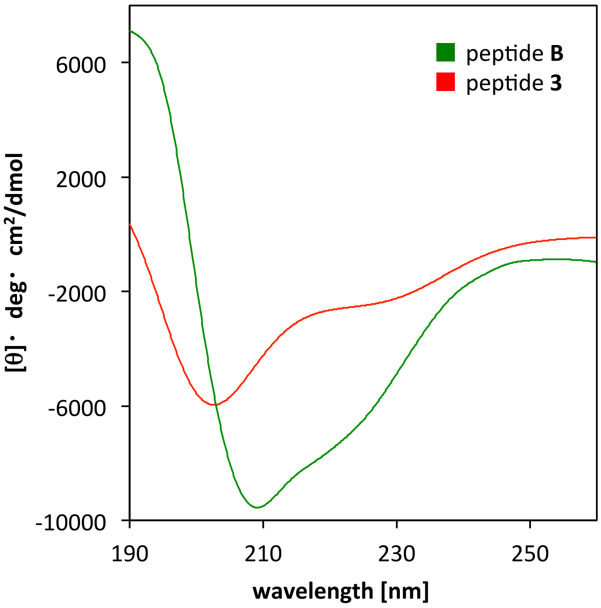

Figure S7. CD spectra of peptides **B** and **3** in 1.0 v/w% SDS in phosphate buffer. Peptide concentration: 100 μM.

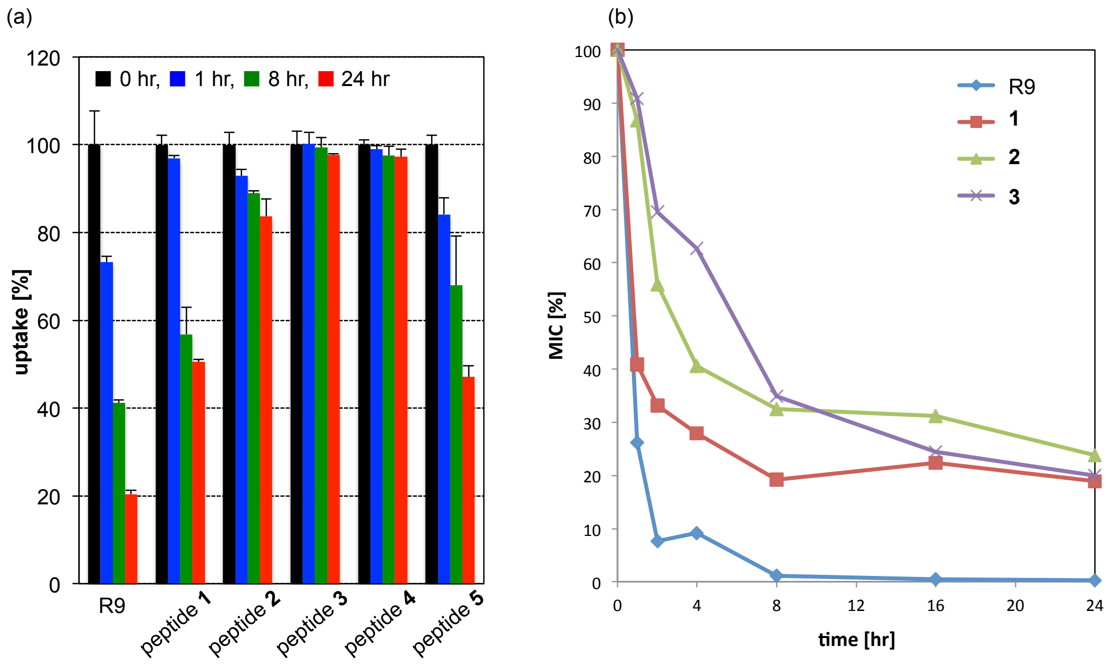

Figure S8. (a) Cellular uptake of the peptides **1-5** after 1-24 h pre-incubation (peptide concentration: 1 μM). Values are the means ± standard deviation of three independent cultures. (b) The peptides' stability (1-24 hr) analysis in culture medium containing FBS using LC-MS.

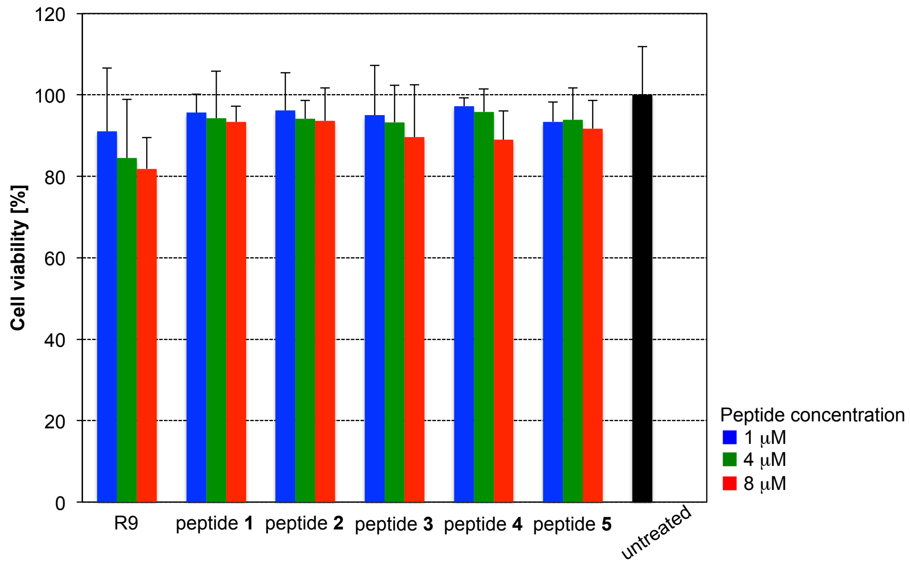

Figure S9. The cytotoxicity of R9 and peptides **1**—**5**.

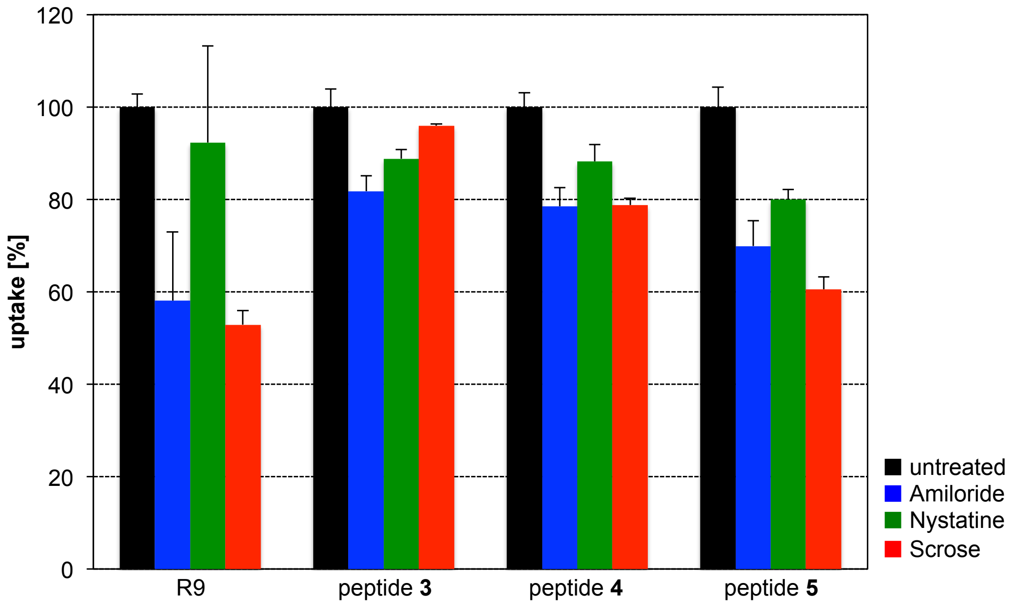

Figure S10. Flow cytometry profile of HeLa cells treated with various endocytosis inhibitors (amiloride, nystatin, scrose).

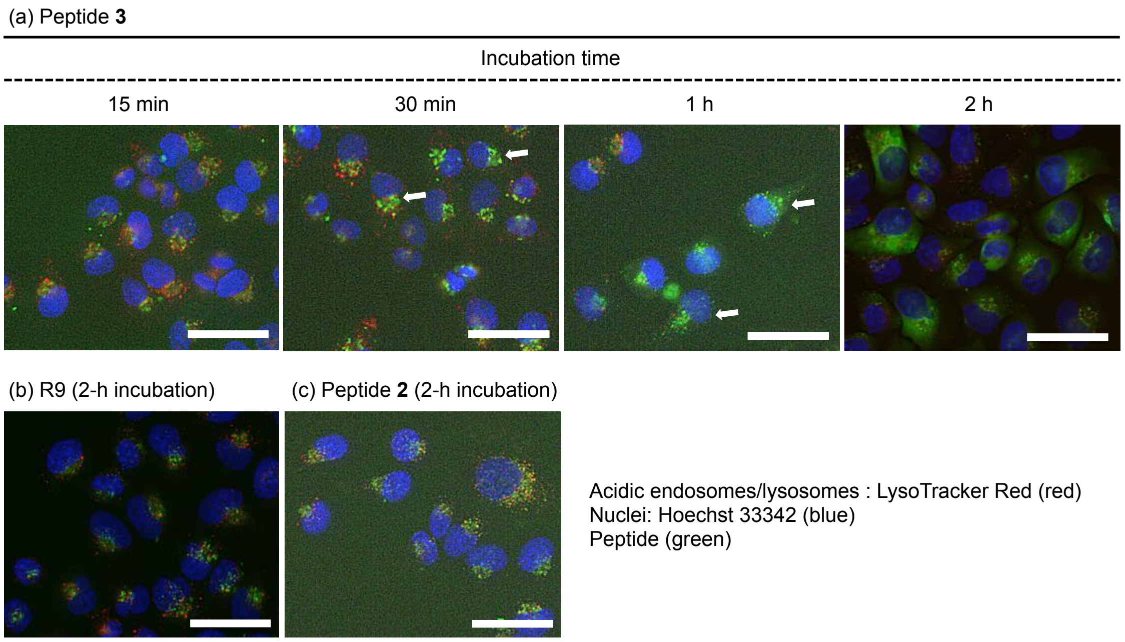

Figure S11. Fluorescence microscopy images of HeLa cells that had been treated with 10 μM of peptides [(a) peptide **3**, (b) R9, (c) peptide **2**] for 15 min-2 hr at 37 °C. The

peptides fluoresced in the cells (green). The acidic late endosomes/lysosomes were stained with LysoTracker Red (red), and the nuclei were stained with Hoechst 33342 (blue). The scale bars represent 50 μm.

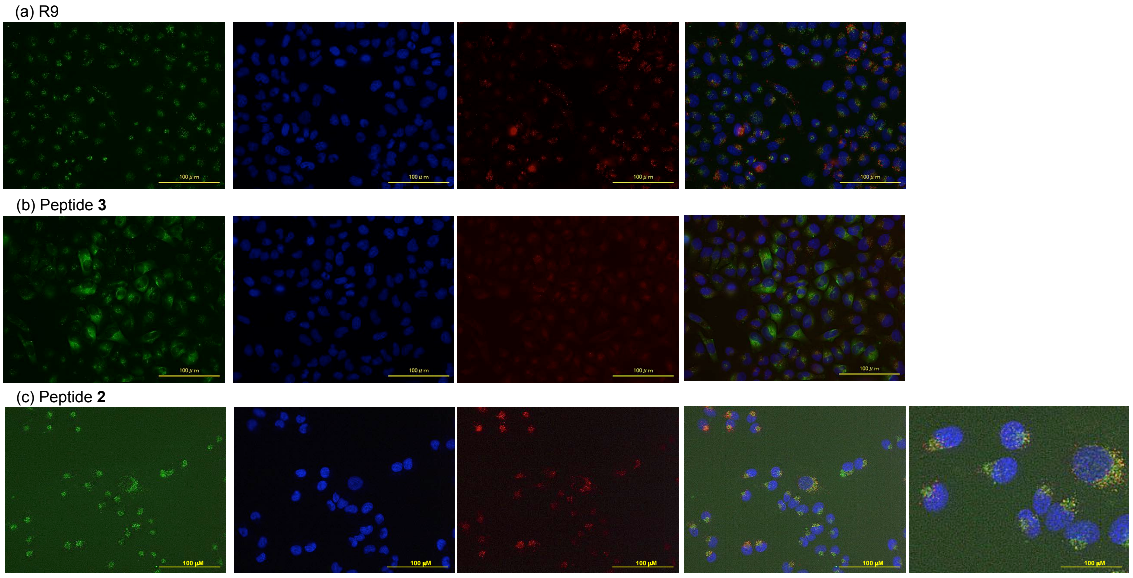

Figure S12. Fluorescence microscopy images of HeLa cells that had been treated with 10 μM of peptides [(a) R9, (b) peptide **3**, (c) peptide **2**] for 2 hr at 37 °C. The peptides fluoresced in the cells (green). The acidic late endosomes/lysosomes were stained with LysoTracker Red (red), and the nuclei were stained with Hoechst 33342 (blue).

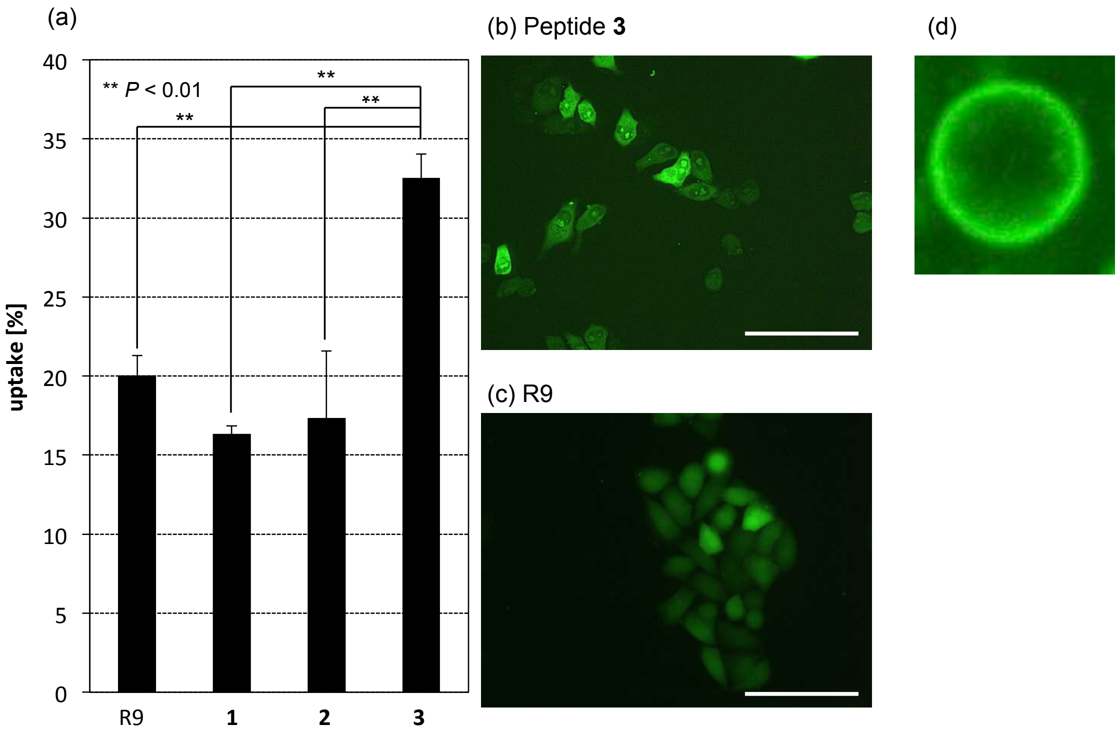

Figure S13. Direct cell permeability of R9 and peptides **1**—**3**. (a) Flow cytometry profile of HeLa cells incubated at 4 °C for 1 hr. 100% uptake was calculated from the fluorescence intensity in the cells incubated at 37 °C. (b), (c) Fluorescence microscopy images of HeLa cells treated with 10-μM peptide **3** (b) and R9 (c) for 30 min at 4 °C. The peptides fluoresced in the cells (green). The scale bars represent 100 μm. (d) Confocal microscopy analysis of GUVs 5 min after the addition of 1 μM peptide **3**. Diameters of GUVs typically observed are about 20 μm.
